# Ring finger protein 213 c.14576G>A mutation is not involved in internal carotid artery and middle cerebral artery dysplasia

**DOI:** 10.1038/s41598-021-01623-6

**Published:** 2021-11-12

**Authors:** Yasuo Murai, Eitaro Ishisaka, Atsushi Watanabe, Tetsuro Sekine, Kazutaka Shirokane, Fumihiro Matano, Ryuta Nakae, Tomonori Tamaki, Kenta Koketsu, Akio Morita

**Affiliations:** 1 Department of Neurological Surgery, Nippon Medical School, Bunkyo-ku, Tokyo, 113-8603 Japan; 2 Division of Clinical Genetics, Kanazawa University Hospital, Kanazawa, Ishikawa 920-8604 Japan; 3 Support Center for Genetic Medicine, Kanazawa University Hospital, Kanazawa, Ishikawa 920-8604 Japan; 4 Department of Radiology, Nippon Medical School Musashi-Kosugi Hospital, Kanagawa, 211-8533 Japan; 5 Department of Emergency and Critical Care Medicine, Nippon Medical School Hospital, Tokyo, 113-8602 Japan; 6 Department of Neurosurgery, Nippon Medical School Tama Nagayama Hospital, Tokyo, 206-8152 Japan

**Keywords:** Genetics, Anatomy, Medical research, Oncology, Risk factors, Signs and symptoms

## Abstract

The ring finger protein 213 (*RNF213*) susceptibility gene has been detected in more than 80% of Japanese and Korean patients with moyamoya disease (MMD), a bilateral internal carotid artery (ICA) occlusion. Furthermore, *RNF213* has been detected in more than 20% of East Asians with atherosclerotic ICA stenosis. In this study, we evaluated the frequency of *RNF213* mutations in congenital occlusive lesions of the ICA system. This case series was conducted jointly at four university hospitals. Patients with a family history of MMD, quasi-MMD, or related diseases were excluded. Ten patients were diagnosed with abnormal ICA or middle cerebral artery (MCA) angiogenesis. Patients with neurofibromatosis were excluded. Finally, nine patients with congenital vascular abnormalities were selected; of these, five had ICA deficiency and four had twig-like MCA. The *RNF213* c.14576G > A mutation was absent in all patients. Therefore, the *RNF213* c.14576G > A mutation may not be associated with ICA and MCA congenital dysplasia—rare vascular anomalies making it difficult to study a large number of cases. However, an accumulation of cases is required for accurate determination. The results of this study may help differentiate congenital vascular diseases from MMD.

## Introduction

Moyamoya disease (MMD) is a progressive, bilateral occlusive disease of the internal carotid artery (ICA) system that eventually leads to stroke. It is more common among Asians, with an incidence of 3–10.5 per 100,000 individuals^1^. MMD diagnosis is based on stenosis or occlusion of the bilateral terminal portions of the ICAs^1^. MMD is a hereditary disease with childhood onset. Other less frequently detected congenital anomalies of angiogenesis, such as ICA deficiency, ICA dysplasia, and twig-like middle cerebral artery (MCA), are also potential causes of stroke^2,3^. ICA deficiency is a rare congenital vascular anomaly that occurs in less than 0.01% of autopsies^4^. MMD and congenital vascular occlusive abnormalities are identified in childhood, whereas atherosclerotic changes are observed in elderly patients. Therefore, elderly patients may have a combination of lesions that make it difficult to differentiate between them or to identify complications^1^.

With recent advances in genetic analysis techniques, genetic differential diagnosis, appropriate risk assessment, prediction of disease onset, and selection of appropriate treatment for cerebrovascular diseases may be possible^5–7^. Moreover, it may lead to the establishment of new diagnostic criteria and disease concepts. Ring finger protein 213 (*RNF213*) has been identified as a susceptibility gene for MMD, and a single missense mutation in *RNF213* (c.14576G > A, p. R4859K, rs112735431) occurs in more than 78% of East Asian patients with MMD^5,7,8^. In familial MMD, it is detected in more than 91% of the patients^9^. *RNF213* is also expressed in 1.8%–4.1% of the normal Asian population without intracranial vascular lesions and in approximately 22%–24% of patients with atherosclerotic occlusive lesions of the ICA system^5,8^. In contrast, it is less frequent in occlusive lesions of the vertebral arteries^8^. However, the relationship between the *RNF213* mutation and congenital vascular dysplasia of the ICA system remains unknown. The aim of this study was to determine the frequency of *RNF213* mutations in congenital occlusive lesions of the ICA system by strict selection based on radiological imaging, previous diseases, and family history.

## Materials and methods

### Data availability

The data that support the findings of this study are available from the corresponding author upon reasonable request.

### Patient selection

This was a case series study conducted in four hospitals affiliated to our university. The study was approved by the Ethics Committee of Nippon Medical School Hospital (Approval Number: H30-26-02). Written informed consent for inclusion in the study, blood sampling, DNA storage, and genetic analysis was obtained from all participants. The study involved patients diagnosed with congenital ICA to MCA occlusive stenotic lesions who visited our hospital between August 2014 and August 2018. As the frequency of the RNF213 c.14576G > A mutation has already been established in Japanese patients with MMD^5,8^, we performed genetic analysis to determine whether the RNF213 c.14576G > A mutation is involved in patients diagnosed with congenital dysplasia of the ICA and MCA. Patients were strictly selected through radiological imaging, as well as medical and family history. Information on sex, age at diagnosis, symptoms, previous diseases, and presence of lifestyle-related diseases was obtained from all patients. The diagnosis of congenital dysplasia of the ICA or MCA was made by at least three experts: two neurosurgeons and a radiologist. We did not include patients diagnosed only by magnetic resonance imaging and angiography or three-dimensional computed tomography. We included those who underwent cerebral angiography as a clinical necessity; however, it was not performed specifically for this study. Patients with abnormalities in the formation of the anterior cerebral artery were excluded. Patients were also excluded if more than one physician found it difficult to differentiate congenital occlusive lesions of the ICA and MCA from MMD or atherosclerotic occlusive disease based on the imaging results.

### Excluded diseases and family history

Patients with MMD or a family history of MMD were excluded^1^. We also excluded patients with a history or family history of diseases that have been linked to MMD^1^. These diseases^1^ included systemic lupus erythematosus, antiphospholipid antibody syndrome, periarteritis nodosa, Sjögren’s syndrome, meningitis, von Recklinghausen’s disease, brain tumors, Down’s syndrome, head trauma, irradiation, hyperthyroidism, narrow head syndrome, Turner’s syndrome, Alagille’s syndrome, Williams syndrome, Noonan syndrome, Marfan syndrome, tuberous sclerosis, Hirschsprung disease, Prader-Willi syndrome, Wilms tumor, primary oxalosis, sickle cell anemia, Fanconi anemia, spherocytosis, eosinophil granuloma, type 2 plasminogen abnormalities, leptospirosis, pyruvate kinase deficiency, protein S deficiency, protein C deficiency, fibromuscular hyperplasia, osteogenesis imperfecta, polycystic kidney disease, oral contraceptives, and drug (e.g., cocaine) addiction. However, we did not exclude patients with atherosclerosis and diabetes mellitus, as these two diseases have been linked to MMD. In addition, patients with pulmonary hypertension, peripheral pulmonary artery stenosis, and coronary artery disease^10^ were excluded from the study, as these diseases have been implicated in *RNF213*^10,11^.

### DNA extraction and RNF213 genotyping

Genomic DNA was extracted from blood using the GENOMIX Kit (Talent, Trieste, Italy). Screening for the genotype of *RNF213* c.14576G > A (exon 61) was performed by small amplicon genotyping based on high resolution melting curve analysis^12^ and confirmed by Sanger sequencing. Polymerase chain reaction primers for c.14576G > A were designed to flank the mutation, leaving only one base containing the mutation between the primers. The forward primer used was 5ʹ-GCAAGTTGAATACAGCTCCATCA-3ʹ and the reverse primer was 5ʹ-TGTGCTTGCTGAGGAAGCCT-3ʹ. Polymerase chain reaction conditions were as follows: initial denaturation at 95 °C for 2 min, followed by 45 cycles at 94 °C for 30 s and annealing at 67 °C for 30 s. Subsequently, 96-well plates were used for high-resolution melting curve analysis using LightScanner (Idaho Technology, Salt Lake City, UT, USA); data were collected data from 55 °C to 97 °C at a ramp rate of 0.101 °C/s.

### Ethics approval

All procedures performed in studies involving human participants were in accordance with the ethical standards of the institutional research committee and with the 1964 Helsinki declaration and its later amendments or comparable ethical standards.

### Consent to participate

Informed consent was obtained from all individual participants included in the study.

### Consent for publication

All authors have read and approved the submitted manuscript. The manuscript has not been submitted nor published elsewhere.

## Results

**Figure 1:**
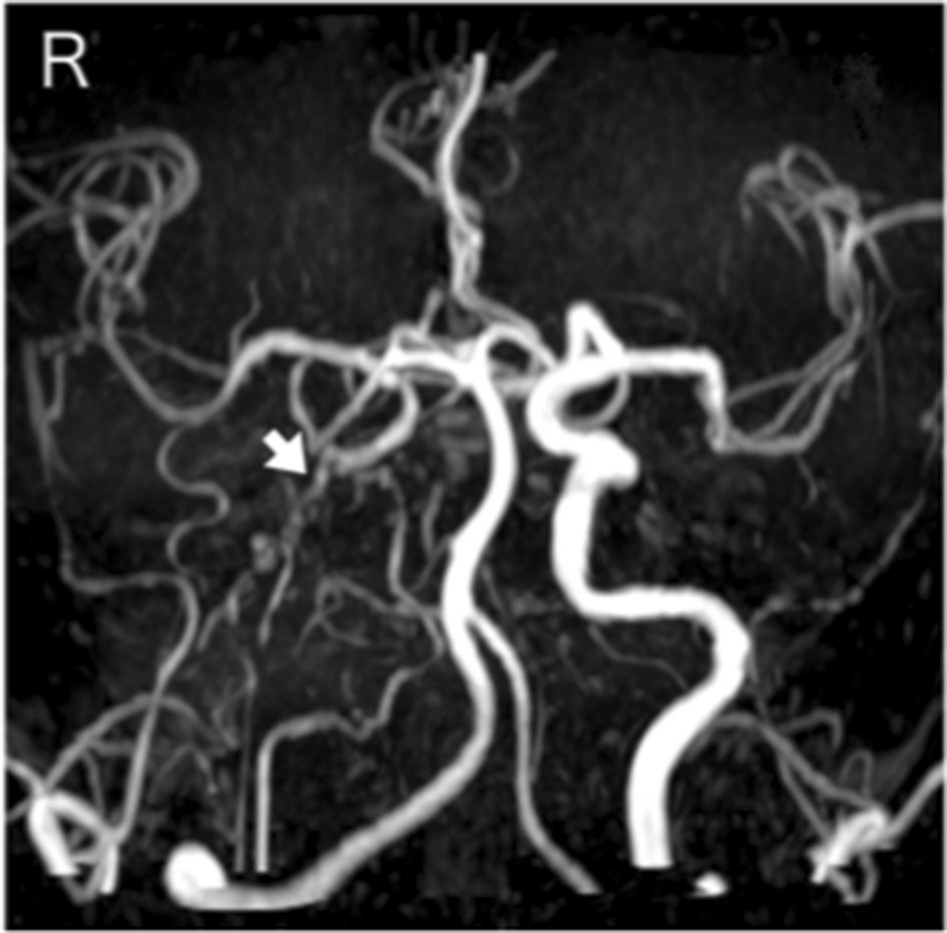
Anterior posterior view of magnetic resonance angiography findings in patient 1 indicating hypoplasia of the right internal carotid artery (white arrow).

**Figure 2:**
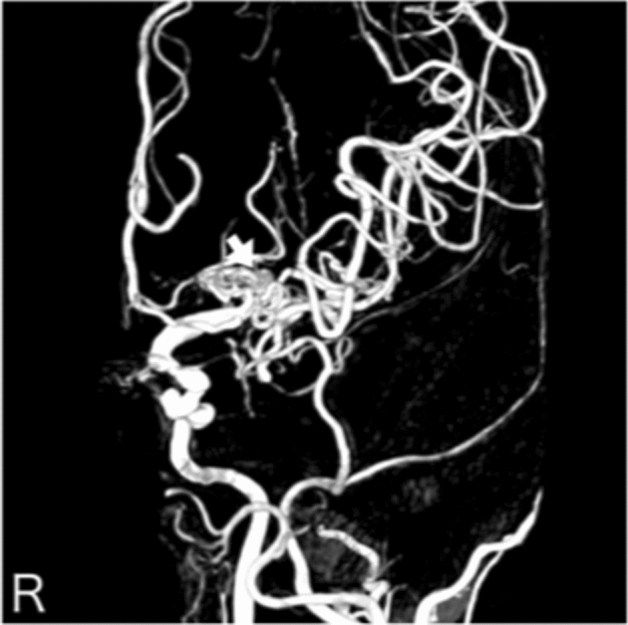
Anterior posterior view of three-dimensional digital subtraction left internal carotid angiographical findings in patient 2 indicating left twig-like middle cerebral artery (white arrow).

**Figure 3:**
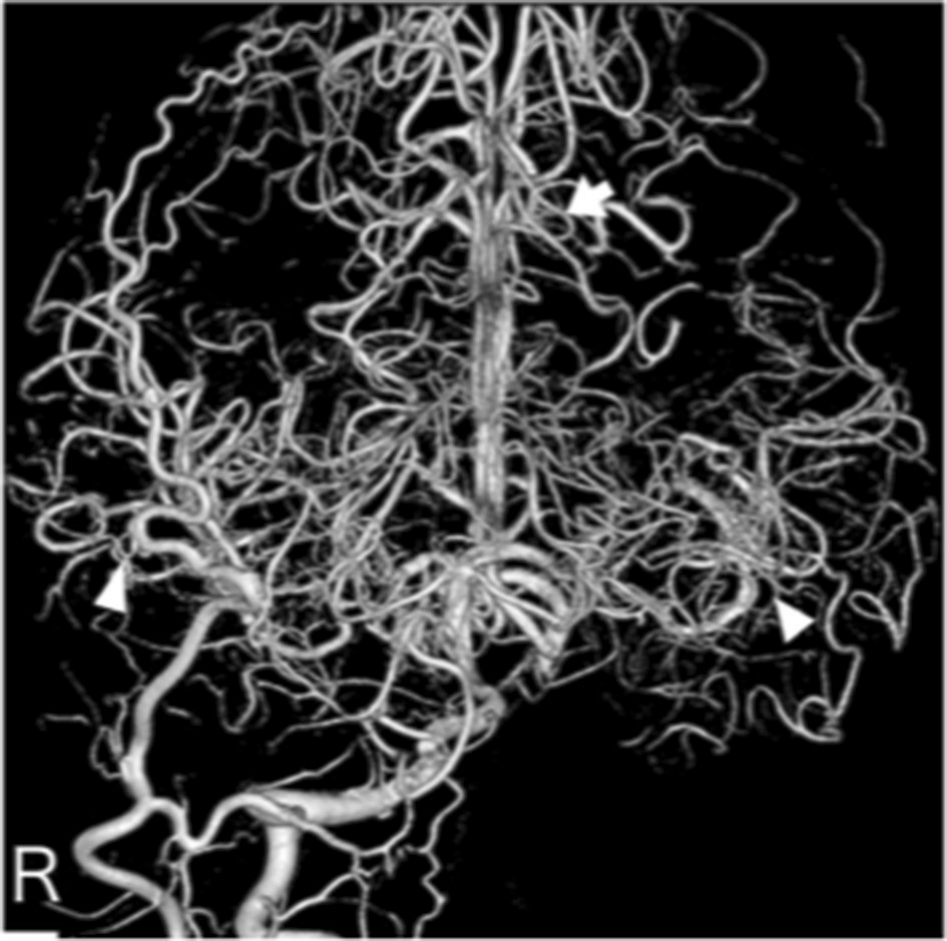
Anterior posterior view of three-dimensional digital subtraction right vertebral angiographical findings in patient 3 indicating the anterior cerebral artery (white arrow) and bilateral middle cerebral artery (white arrow heads) flow from the posterior communicating artery.

**Figure 4:**
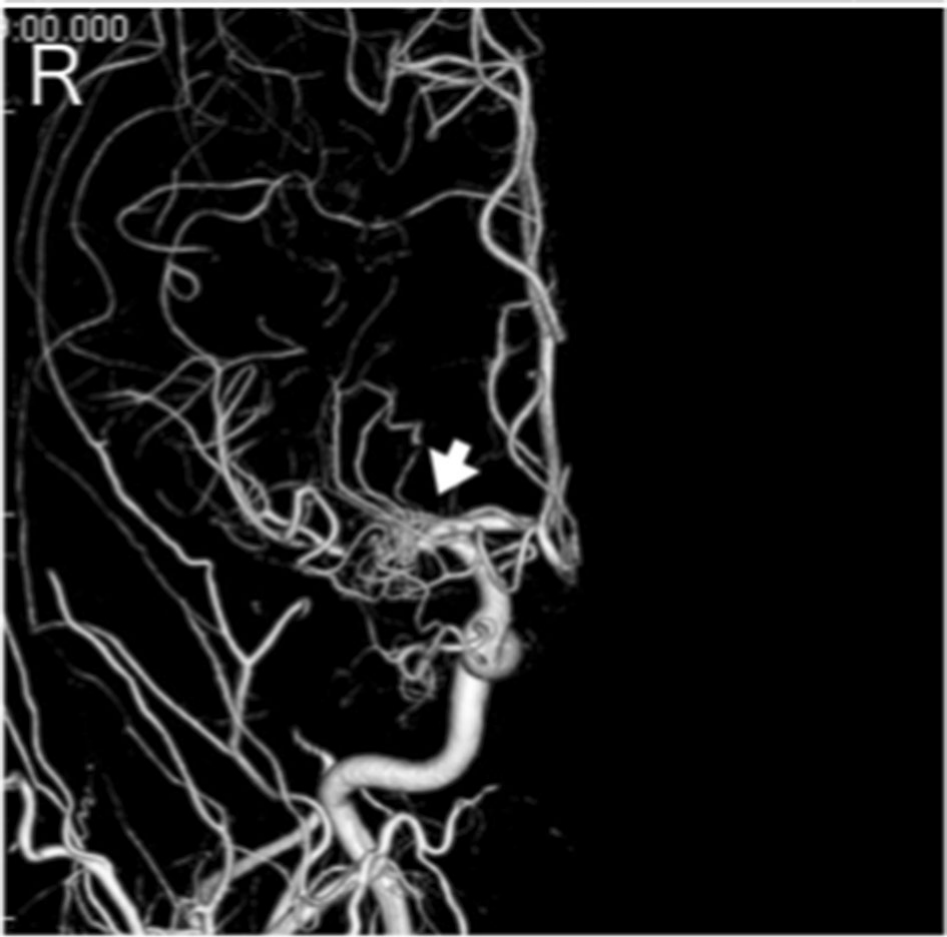
Anterior posterior view of three-dimensional digital subtraction right internal carotid angiographical findings in patient 4 indicating right twig-like middle cerebral artery. There was no stenotic area and moyamoya vessels in the terminal portion of the internal carotid artery (white arrow). These imaging findings did not indicate moyamoya disease.

**Figure 5:**
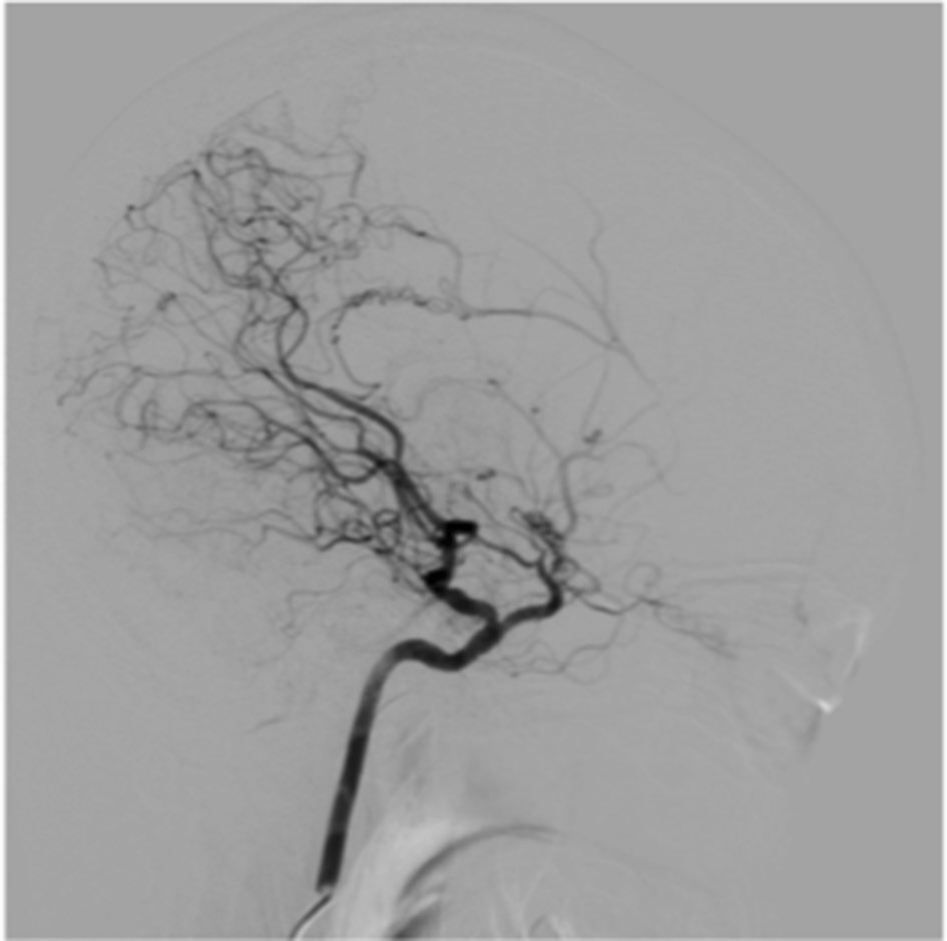
Lateral view of left digital subtraction internal carotid angiographical findings in patient 5 indicating primitive trigeminal artery and right middle cerebral artery hypoplasia.

**Figure 6:**
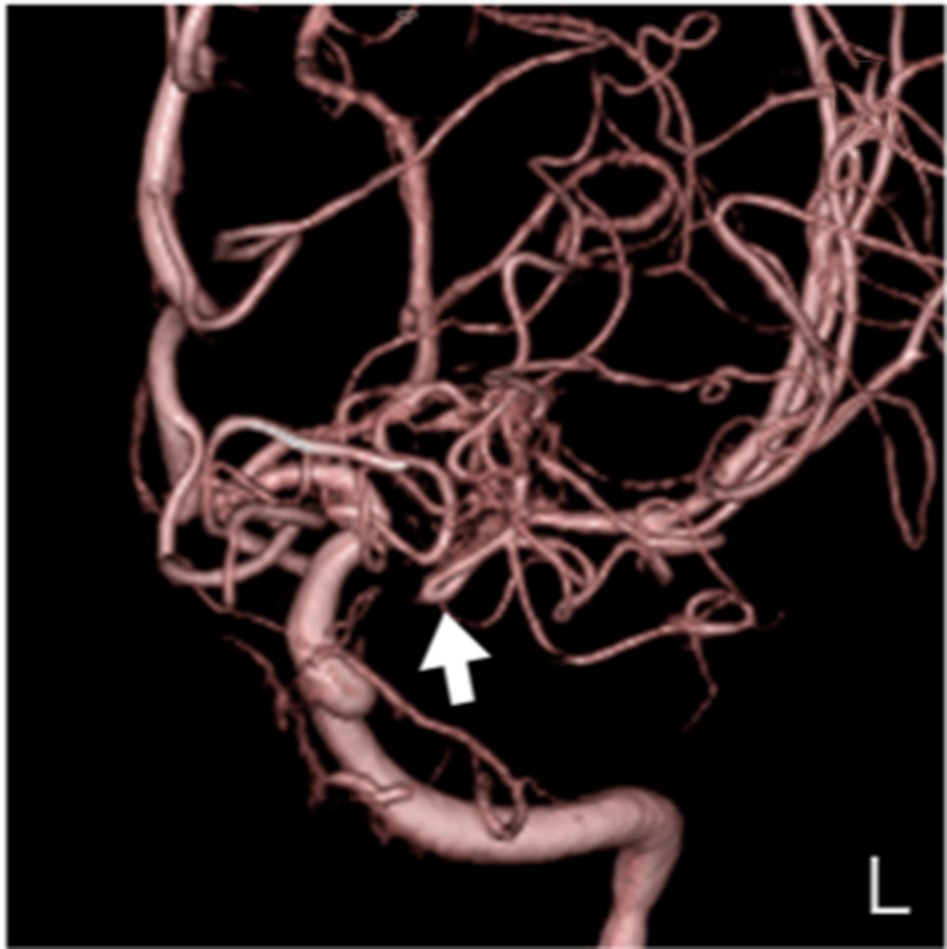
Anterior posterior view of three-dimensional digital subtraction left internal carotid angiographical findings in patient 6 indicating left twig-like middle cerebral artery (white arrow). The moyamoya vessels were not present.

**Figure 7:**
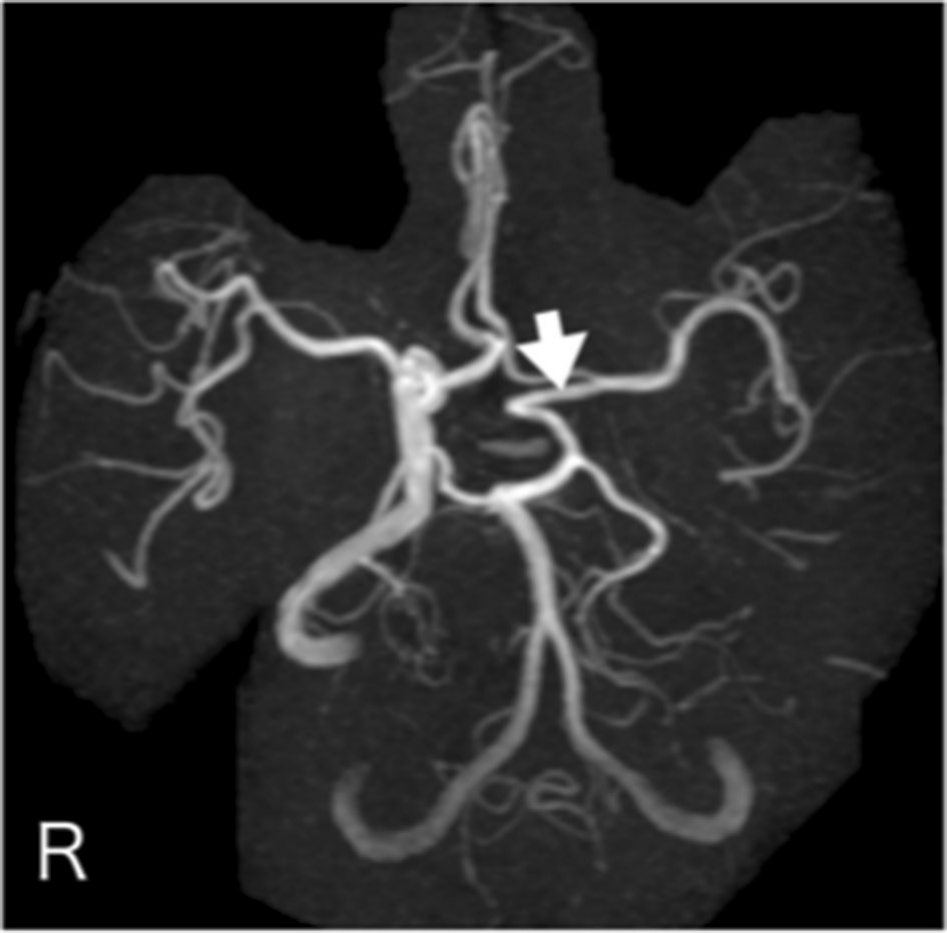
Anterior posterior view of magnetic resonance angiography findings in patient 7 indicating aplasia of the left internal carotid artery (white arrow).

**Figure 8:**
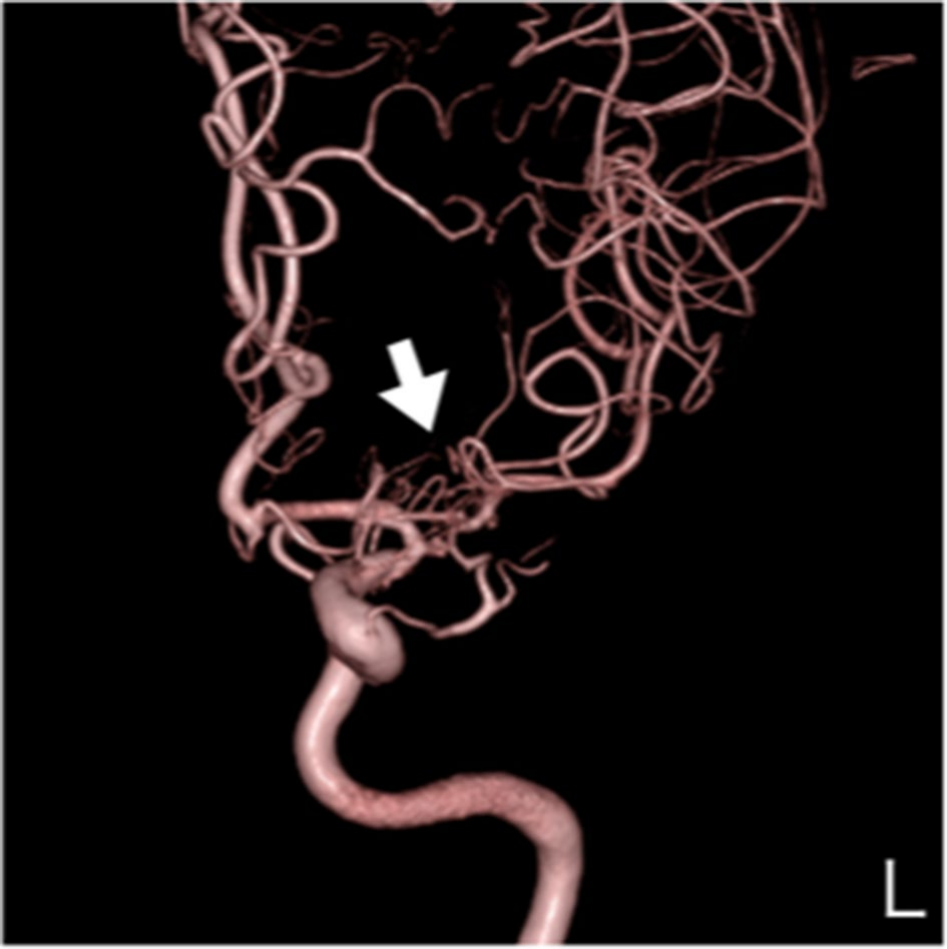
Anterior posterior view of three-dimensional digital subtraction left internal carotid angiographical findings in patient 8 indicating left twig-like middle cerebral artery (white arrow). The moyamoya vessels were not present.

**Figure 9:**
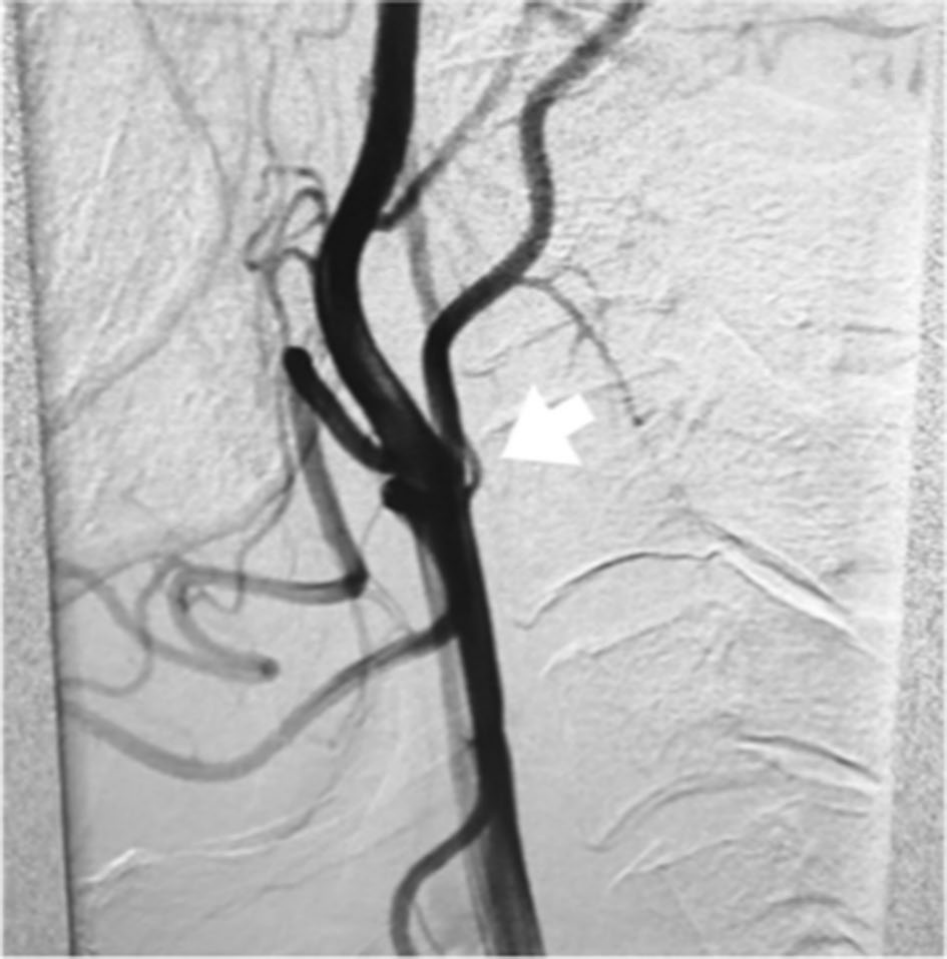
Lateral view of digital subtraction left cervical common carotid angiographical findings in patient 9 indicating aplasia of the left internal carotid artery (white arrow).

Among the patients diagnosed with congenital vascular anomalies after angiographic imaging during the study, one was excluded because of concomitant neurofibromatosis and another for concomitant brain tumor^1^. In total, nine patients (five women and four men) were eventually enrolled during the study. The clinical characteristics of all patients are shown in Table. The median age (interquartile range) at diagnosis was 53 (26.5–62) years. The type of onset was cerebral ischemia in three patients, headache in two, dizziness in one, intracranial hemorrhage in one, and asymptomatic in two patients. Cerebrovascular radiological images of all patients are presented in Figs. 1–9 (supplemental materials). The two genotypes of *RNF213*, c.14576G > A, that is, c.14576GG (wt/wt) and c.14576AG (mut/wt), were determined using the modified small amplicon genotyping method for all patients. The c.14576G > A and A > A mutations in *RNF213* were absent in all patients.

Additional figures for all cases are included in the supplemental material.

### Case presentation

Additional figures for all cases are included in the supplemental material.

**Case 1:** A male in his 50 s underwent magnetic resonance imaging (MRI) and angiography (Fig. 1) for vertigo, and they indicated a right internal carotid artery occlusion. There was no family history of cerebral infarction, cerebral hemorrhage, or intracranial disease. Forty years ago, the patient had undergone cerebral angiography, and the doctor in charge at that time explained that one large blood vessel was missing. The findings of time-of-flight magnetic resonance angiography axial source imaging (Supplement Fig. 1-1) indicated dysplastic internal carotid artery in the right cavernous sinus. Coronal MRI showed corpus callosum dysplasia (Supplement Fig. 1-2).

**Case 2:** A woman in her 60 s was referred to our hospital because she was suspected to have left middle cerebral artery occlusion or unilateral moyamoya disease based on MRI and MRA performed for a thorough headache examination. There was no family history of cerebral infarction, cerebral hemorrhage, or intracranial disease. Cerebral angiography showed multiple “fenestrated like vessels in the M1 area” only on the left side, and no moyamoya vessels (Fig. 2). The right posterior communicating artery was of fatal type (Supplement Fig. 2-1). Evaluation of cerebral blood flow using I-123 single-photon emission computed tomography showed only a mild decrease in blood flow (Supplement Fig. 2-2), and the patient is under observation without any special treatment.

**Case 3:** A man in his 20 s presented with a headache and was diagnosed with subarachnoid hemorrhage by computed tomography. There was no history indicating atherosclerotic changes. Cerebral angiography showed bilateral internal carotid artery dysplasia (Fig. 3 and Supplemental Fig. 3-1 and 2). The right and left middle cerebral arteries and anterior cerebral arteries were delineated from the vertebral arteries through the bilateral posterior traffic arteries (Fig. 3 and Supplemental Fig. 3-1). The left vertebral artery was also stenotic (Supplemental Fig. 3-1). No cerebral aneurysm was identified to suggest a cause of subarachnoid hemorrhage.

**Case 4:** A woman in her 50 s was referred to our hospital because she was suspected of having right middle cerebral artery stenosis or unilateral moyamoya disease by MRI and MRA, which were performed for a thorough examination of headache. There was no family history of cerebral infarction, cerebral hemorrhage, or intracranial disease. Cerebral angiography showed fenestrated blood vessels in the M1 area only on the right side, but no moyamoya vessels and a diagnosis of Twig-like MCA was made (Fig. 4 and Supplemental Fig. 4-1 and 2). MRI also showed no moyamoya vessels in the basal ganglia (Fig. 4 and Supplemental Fig. 4-2).

**Case 5:** A woman in her 20 s was referred to our hospital with a diagnosis of cerebral infarction after a thorough examination of transient left hemiparalysis. She had no family history of cerebral infarction, cerebral hemorrhage, or intracranial disease. Cerebral angiography showed a primitive trigeminal artery on the right, and the internal carotid artery was defective at its terminus (Fig. 5). Neither cerebral angiography nor MRI showed any moyamoya vessels (Fig. 5 and supplemental Fig. 5-1). The right internal carotid artery territory flowed by collateral pathways from the vertebral and left internal carotid arteries. Her carotid angiogram indicated no stenotic lesions in the left internal carotid artery (Supplemental Fig. 5-2).

**Case 6:** A woman in her 40 s was referred to our hospital because she was suspected to have left middle cerebral artery stenosis by MRI and MRA, which were performed for a physical examination. She had no family history of cerebral infarction, cerebral hemorrhage, or intracranial disease. Cerebral angiography showed fenestrated vessels in M1 only on the left side, no moyamoya vessels, and good visualization of M2 to M5, leading to the diagnosis of Twig-like MCA (Fig. 6 and Supplemental Fig. 6-1). There were no occlusive changes in the internal carotid artery and middle cerebral artery on the right, and no moyamoya vessels in the basal ganglia were observed on MRI (Supplemental Fig. 6-1, 2).

**Case 7:** A woman in her 60 s was referred to our hospital because she was suspected to have left internal carotid artery occlusion based on MRI and angiography performed to find the cause of her headache (Fig. 7). There was no family history of cerebral infarction, cerebral hemorrhage, or intracranial disease. Axial computed tomography (Supplemental Fig. 7-1) showed no carotid canal on the left, and cervical magnetic resonance angiography (Supplemental Fig. 7-2) showed the left internal carotid artery was aplastic from the cervical carotid bifurcation.

**Case 8:** A woman in her 60 s presented with right hemiparalysis and was referred to our hospital with suspected left caudate nucleus cerebral infarction and left middle cerebral artery occlusion based on MRI and angiography. There was no family history of cerebral infarction, cerebral hemorrhage, or intracranial disease. Cerebral angiography showed multiple fenestrated blood vessels in the M1 region only on the left side and no moyamoya vessels (Fig. 8). On MRI, no abnormal moyamoya vessels were observed in the basal ganglia (Supplemental Fig. 8-1). There were no occlusive changes in the internal carotid artery and middle cerebral artery on the right (Supplemental Fig. 8-2).

**Case 9:** A man in his 50 s presented with transient right hemiparesis and was referred to our hospital with suspected left internal carotid artery occlusion based on MRI and angiography (Supplemental Fig. 9-1). He had no family history of cerebral infarction, cerebral hemorrhage, or intracranial disease. On cerebral angiography, the left internal carotid artery was missing from the neck (Fig. 9), and the middle cerebral artery was flowing from the vertebral artery through the posterior communicating artery. There were no occlusive changes in the right internal carotid artery or middle cerebral artery. MRI also showed no left internal carotid artery scarring in the cavernous sinus (Supplemental Fig. 9-2).

## Discussion

Our findings showed that the c.14576G > A mutations in *RNF213* did not occur in any of the nine patients diagnosed with congenital ICA and MCA vascular abnormalities after rigorous radiological imaging and family and medical history investigation. Thus, as this was a case series study, the possibility of *RNF213* mutations was lower than that of MMD. However, the number of cases could not be sufficiently accumulated for examination.

Because of the low detection rate of these congenital anomalies, only a few cases have been reported to date. *RNF213* mutations, detected in more than 80% of Japanese patients with MMD and approximately 20% of patients with atherosclerotic stenosis, are unlikely to be identified with the same frequency in these congenital anomalies^5,8^. Furthermore, the *RNF213* mutations are detected in a higher proportion of familial lesions (90%) than in solitary cases (> 80%)^9^. Therefore, patients with a family history of MMD were excluded from this study. The results of this study may help in the differential diagnosis of ICA lesions; however, more cases are needed to validate the present findings.

*RNF213*, suspected of having a genetic component owing to its familial occurrence and high prevalence in East Asian populations, was reported in 2011^9,13^. In a case–control association study, p.Glu4950Asp was reported as the dominant variant in Koreans^14^, and p.Ala4399Thr, p.Glu4950Asp, and p.Ala5021Val were reported as the dominant variants in Chinese^7,15^. However, no dominant variants have been reported in Japanese. The analysis of p.Arg4810Lys has shown that approximately 25% of the diagnosed intracranial atherosclerotic stenosis do not meet the diagnostic criteria for MMD^5^. Furthermore, the site of stenosis has been characterized, with the ICA and MCA being more frequent than the vertebrobasilar artery^8^. It has been suggested that this difference in the embryological background might affect the smooth muscles of the vertebrobasilar artery system of the posterior circulation, although the smooth muscles of the ICA vasculature from the petrous portion to the periphery are derived from the neural crest^16^. Recently, *RNF213* p.Arg4810Lys has been associated with pulmonary artery stenosis^17^, coronary artery^10^ stenosis, and renal vessel stenosis, and the fact that these vessels are of neural crest origin also supports the opinion^11^.

Although there are several reports on the *RNF213* polymorphism as a susceptibility gene for MMD, we focused on *RNF213* c.14576G > A, (p.R4859K, rs112735431) in the present study. In a report^18^ on surveying research trends in MMD up to 2019, "c.14576" was extracted as the top-cited keyword, as the only number indicating a polymorphism in the *RNF213* gene. The other reasons for this are as follows. First, *RNF213*, a susceptibility gene for MMD first reported in Japan in 2011, was reported from two completely different institutions^9,13^. Both were *RNF213* c.14576G > A (p.R4859K, rs112735431).　Subsequent studies^7,19–23^ have also reported the *RNF213* polymorphism in MMD, but there is a bias by country^20^. In fact, p.R4810K^22^, which has garnered attention as a susceptibility gene for MMD in East Asians other than p.R4859K, has been reported from Japan^24^, China^22,25,26^, and Korea^27^. Overall, the pooled results^19^ indicated that both p.R4810K and p.R4859K are associated with an MMD risk (OR reported to be associated with an MMD risk (OR 92.03, 95% CI 54.06–156.65, P < 0.00001 and OR 157.53, 95% CI 85.37–290.7, P < 0.00001, respectively)^19^. Furthermore, we focused on these p.R4859K polymorphisms^28^ as susceptibility genes for systemic vascular diseases. The p.R4859K polymorphisms have been reported in Japanese patients with combined MMD and peripheral pulmonary artery stenosis^29^, pulmonary vasculopathy^17^, cerebral aneurysm development^30^, CADASIL^31^, and reversible cerebral angiopathy^32^.

Twig-like MCA is described as a disease in which the unilateral M1 is not depicted on cerebral angiography, and the surrounding collateral arteries are developed (spontaneous MCA occlusion with moyamoya phenomenon [MCAO-Mo])^3,6^. Recently, these conditions have been regarded as disruptive collateral circulation due to the failure of fusion and retraction of the primitive reticular vasculature in M1, and have been proposed as "aplastic or twig-like MCA (Ap/T-MCA)" by Seo et al.^3^ Ap/T-MCA is a rare vascular malformation with a frequency of 0.11–1.17%^3^. Twig-like MCA, similar to the MCA window formation, is caused by a portion of the vascular network remaining during the differentiation of the MCA window formation at 6 mm (4–5 weeks of fetal life). There is a paucity of research on the relationship between these congenital vascular lesions and MMD, both an occlusive disease of the ICA and MCA. However, a 53-year-old woman with ruptured MCA peripheral multiple aneurysms associated with twig-like MCA and *RNF213* gene mutation has been reported^6^. The patient had suffered a stroke once at a relatively young age, in her early 40 s, and a family history of cerebral infarction was noted. Therefore, there was a possibility of a family history of MMD. However, except this study, we could not find any study on the relationship between congenital anomalies of angiogenesis and *RNF213* gene mutation.

There are various reports on the relationship between MMD and persistent primitive arteries. The coexistence of moyamoya and residual vessels is more frequent in Asians than in other populations. In a study of 50 patients with MMD, including quasi-MMD, anastomoses of the persistent primitive carotid-basilar artery were observed in three patients: the primitive sublingual artery in one patient with MMD, a variant of the primitive trigeminal artery in one patient with unilateral MMD, and anastomoses of the accessory meningeal artery and the anterior superior cerebellar artery in one patient with quasi-MMD. The ophthalmic artery originated from the middle meningeal artery in three patients with MMD and two with quasi-MMD. In other words, the incidence of persistent primitive arteries was 10.7% in MMD patients and 60% in quasi-MMD patients. However, a German study found only one patient with PPTA (0.89%) among 122 patients with MMD (112 patients) and quasi-moyamoya syndrome (10 patients). Thus, although the relationship between angioplasty abnormalities and MMD, which are believed to be caused in utero, has not been studied coherently due to the small number of patients, the results of this study did not show a high hereditary association.

Thus, a congenital vascular abnormality is due to fetal factors and therefore unrelated to *RNF213*, whereas diseases related to neural crest may be associated with *RNF213.*

### Statistically necessary samples

The study was conducted over 4 years, but we could gather only 9 patients, and all of them were negative for *RNF213*. For future case control studies, we will consider the sample size needed for statistical studies. We assumed that the comparison subjects for the case–control study would be MMD for which multiple reports reported a frequency of *RNF213* greater than 84%^13,28,33^, or atherosclerotic intracranial internal carotid artery stenotic disease, for which a frequency of *RNF213* greater than 20%^5,8^ was reported. We also assumed that the frequency of *RNF213* in the general Japanese population and cerebral aneurysms would be approximately 1.5%^9,33^. From this result, we determined the appropriate sample size and power for statistical analysis. Categorical variables were compared using Fisher’s exact test. A p-value < 0.05 was considered to indicate statistical significance. All analyses were performed using JMP 14.0 software (SAS Institute Inc., Cary, NC). Assuming a type 1 error of 5% (α = 0.05) and a power of 95% (1-β = 0.95), we calculated the sample size required to compare positive cases of moyamoya disease and internal carotid artery dysplasia. Based on recent reports, we assumed that approximately 20% of Japanese patients with atherosclerotic intracranial internal carotid artery stenosis express *RNF213* and that patients with internal carotid artery dysplasia do not express *RNF213*. The number of cases required under these conditions was 56 per group. Next, based on a recent study, we calculated the required sample size assuming that *RNF213* is expressed in 1.5% of the general Japanese population and found that 875 patients per group would be required.

## Advantage and limitations

Our study had some limitations. First, the number of cases is small and additional research is needed. For our study (supplement material), we had to gather more than 56 patients. Second, the patients in this study mostly had intracranial carotid abnormalities that did not develop until adulthood. Thus, this study was biased. In addition, due to technical problems at the beginning of our research plan, we could not conduct a more robust genome-wide association study (GWAS) and exome analysis using next-generation sequencing to identify genetic polymorphisms. This is the next challenge for our research team.

**Table 1:** Demographic and clinical characteristics of patients enrolled in the study.

	Age/sex	Bil/unilateral	Diagnosis	HT	DM	DL	Smoking	Symptom
1	50 s/M	Unilateral	ICA aplasia	(−)	(−)	(−)	( +)	Dizziness
2	60 s/F	Unilateral	Twig-like MCA	( +)	(−)	(−)	(−)	Headache
3	20 s/M	Bilateral	Bilateral ICA dysplasia Bilateral VA dysplasia	(−)	(−)	(−)	(−)	Hemorrhage
4	50 s/F	Unilateral	Twig-like MCA	( +)	(−)	( +)	(−)	Incidental
5	20 s/F	Unilateral	ICA MCA hypoplasia Primitive Trigeminal artery	(−)	(−)	(−)	( +)	Ischemia
6	40 s/M	Unilateral	Twig-like MCA	(−)	(−)	(−)	( +)	Incidental
7	60 s/F	Unilateral	ICA aplasia	(−)	(−)	( +)	( +)	Headache
8	60 s/F	Unilateral	Twig-like MCA	(−)	(−)	(−)	(−)	Ischemia
9	50 s/M	Unilateral	ICA aplasia	(−)	(−)	(−)	(−)	Ischemia

*DL* dyslipidemia, *DM* diabetes mellitus, *F* female, *HT* hypertension, *ICA* internal carotid artery, *M* male, *MCA* middle cerebral artery, *VA* vertebral artery.

Age, age at admission. Case 1 was diagnosed at 9 years old.

## Supplementary Information

Supplementary Information.

## Abbreviations

ICA: Internal carotid artery
MCA: Middle cerebral artery
MMD: Moyamoya disease
*RNF213*: Ring finger protein 213

## References

[1] Guidelines for Diagnosis and Treatment of Moyamoya Disease (Spontaneous Occlusion of the Circle of Willis): Esearch Committee on the Pathology and Treatment of Spontaneous Occlusion of the Circle of Willis; Health Labour Sciences Research Grant for Resear. *Neurol Med Chir (Tokyo)* **52**, 245–266 (2012). 10.2176/nmc.52.245 22870528

[2] Rosen IW, Mills DF, Nadel HI, Kaiserman DD (1975). Angiographic demonstration of congenital absence of both internal carotid arteries—Case report. J. Neurosurg..

[3] Seo BS (2012). Clinical and radiological features of patients with aplastic or twiglike middle cerebral arteries. Neurosurgery.

[4] Smith KR, Nelson JS, Dooley JM (1968). Bilateral, “hypoplasia” of the internal carotid arteries. Neurology.

[5] Miyawaki S (2013). Genetic variant RNF213 c.14576G>A in various phenotypes of intracranial major artery stenosis/occlusion. Stroke.

[6] Fukuyama R (2020). Ruptured aneurysm of an aplastic or twig-like middle cerebral artery with ring finger protein 213 mutation: A case report. No Shinkei Geka.

[7] Liao X, Deng J, Dai W, Zhang T, Yan J (2017). Rare variants of RNF213 and moyamoya/non-moyamoya intracranial artery stenosis/occlusion disease risk: A meta-analysis and systematic review. Environ. Health Prev. Med..

[8] Shinya Y (2017). Genetic analysis of ring finger protein 213 (RNF213) c.14576G>A in intracranial atherosclerosis of the anterior and posterior circulations. J. Stroke Cerebrovasc. Dis..

[9] Liu W (2011). Identification of RNF213 as a susceptibility gene for moyamoya disease and its possible role in vascular development. PLoS ONE.

[10] Morimoto T (2017). Significant association of RNF213 pR4810K, a moyamoya susceptibility variant, with coronary artery disease. PLoS ONE.

[11] Bang OY (2020). Moyamoya disease and spectrums of RNF213 vasculopathy. Transl. Stroke Res..

[12] Watanabe A (2011). Prevalence of c1559delT in ALPL, a common mutation resulting in the perinatal (lethal) form of hypophosphatasia in Japanese and effects of the mutation on heterozygous carriers. J. Hum. Genet..

[13] Kamada F (2011). A genome-wide association study identifies RNF213 as the first Moyamoya disease gene. J. Hum. Genet..

[14] Park YS (2017). The role of RNF213 4810G>a and 4950G>A variants in patients with moyamoya disease in Korea. Int. J. Mol. Sci..

[15] Wu Z (2012). Molecular analysis of RNF213 gene for moyamoya disease in the Chinese Han population. PLoS ONE.

[16] Komiyama M (2018). RNF213 genetic variant and the arterial circle of willis. J. Stroke Cerebrovasc. Dis..

[17] Fukushima H, Takenouchi T, Kosaki K (2016). Homozygosity for moyamoya disease risk allele leads to moyamoya disease with extracranial systemic and pulmonary vasculopathy. Am. J. Med. Genet. Part A.

[18] Chen D (2021). mapping trends in moyamoya angiopathy research: a 10-year bibliometric and visualization-based analyses of the web of science core collection (WoSCC). Front. Neurol..

[19] Ma J (2013). RNF213 polymorphism and Moyamoya disease: A systematic review and meta-analysis. Neurol. India.

[20] Wang X (2020). Association of genetic variants with moyamoya disease in 13 000 individuals: A meta-analysis. Stroke.

[21] Wang Y (2020). Predictive role of heterozygous p.R4810K of RNF213 in the phenotype of Chinese moyamoya disease. Neurology.

[22] Zhang T (2017). Genetic analysis of RNF213 p.R4810K variant in non-moyamoya intracranial artery stenosis/occlusion disease in a Chinese population. Environ. Health Prev. Med..

[23] Zhu B (2020). RNF213 gene polymorphism rs9916351 and rs8074015 significantly associated with moyamoya disease in Chinese population. Ann. Transl. Med..

[24] Shinya Y (2020). Hemorrhagic onset intracranial artery dissection of middle cerebral artery followed by progressive arterial stenosis with genetic variant RNF213 p.Arg4810Lys (rs112735431). World Neurosurg..

[25] Zhang Q (2017). The association of the RNF213 p.R4810K polymorphism with Quasi-Moyamoya disease and a review of the pertinent literature. World Neurosurg..

[26] A Meta-Analysis.Y, W. (2018). RNF213 p. R4810K polymorphism and the risk of moyamoya disease, intracranial major artery stenosis/occlusion, and quasi-moyamoya disease. J. Stroke Cerebrovasc. Dis..

[27] Kim EH (2016). Importance of RNF213 polymorphism on clinical features and long-term outcome in moyamoya disease. J. Neurosurg..

[28] Ishisaka E (2021). Role of RNF213 polymorphism in defining quasi-moyamoya disease and definitive moyamoya disease. Neurosurg. Focus.

[29] Takahashi K (2020). A histopathological report of a 16-year-old male with peripheral pulmonary artery stenosis and Moyamoya disease with a homozygous RNF213 mutation. Respir. Med. Case Rep..

[30] Fukushima Y (2015). Repeated de novo aneurysm formation after anastomotic surgery: Potential risk of genetic variant RNF213 c14576G>A. Surg. Neurol. Int..

[31] Yeung WTE (2018). RNF213-related susceptibility of Japanese CADASIL patients to intracranial arterial stenosis. J. Hum. Genet..

[32] Echizenya I, Tokairin K, Kawabori M, Kazumata K, Houkin K (2020). Reversible cerebral angiopathy after viral infection in a pediatric patient with genetic variant of RNF213. J. Stroke Cerebrovasc. Dis..

[33] Miyawaki S (2012). Identification of a genetic variant common to moyamoya disease and intracranial major artery stenosis/occlusion. Stroke.

