# Supplementary material for: Ring finger protein 213 c.14576G>A mutation is not involved in internal carotid artery and middle cerebral artery dysplasia
Source: Sci Rep. 2021 Nov 12;11:22163. doi: 10.1038/s41598-021-01623-6 (PMC8589854; doi:10.1038/s41598-021-01623-6)
Supplement: Supplementary file 1 — Supplementary Information. [file 41598_2021_1623_MOESM1_ESM.pdf]

Supplemental Materials

Title: Ring Finger Protein 213 c.14576G>A mutation is not involved in internal carotid artery and middle cerebral artery dysplasia

Yasuo Murai, M.D., Ph.D.,[1,*] Eitaro Ishisaka, M.D.,[1] Atsushi Watanabe, M.D., Ph.D.,[2,3] Tetsuro Sekine, M.D., Ph.D.,[4] Kazutaka Shirokane, M.D., Ph.D.,[1] Fumihiro Matano, M.D., Ph.D.[1] Ryuta Nakae, M.D., Ph.D.,[5] Tomonori, Tamaki, M.D., Ph.D.,[6] Kenta Koketsu, M.D., Ph.D.,[1]and Akio Morita, M.D., Ph.D.[1]

[1]Department of Neurological Surgery, Nippon Medical School, Bunkyo-ku, Tokyo, Japan

[2]Division of Clinical Genetics, Kanazawa University Hospital, Kanazawa, Ishikawa, Japan

[3]Support Center for Genetic Medicine, Kanazawa University Hospital, Kanazawa, Ishikawa, Japan

[4]Department of Radiology, Nippon Medical School Musashi-Kosugi Hospital, Kanagawa, 211-8533 Japan

[5]Department of Emergency and Critical Care Medicine, Nippon Medical School Hospital, Tokyo, 113-8602 Japan

[6]Department of Neurosurgery, Nippon Medical School TamaNagayama Hospital, Tokyo, Japan

Supplemental Figures

Case 1:

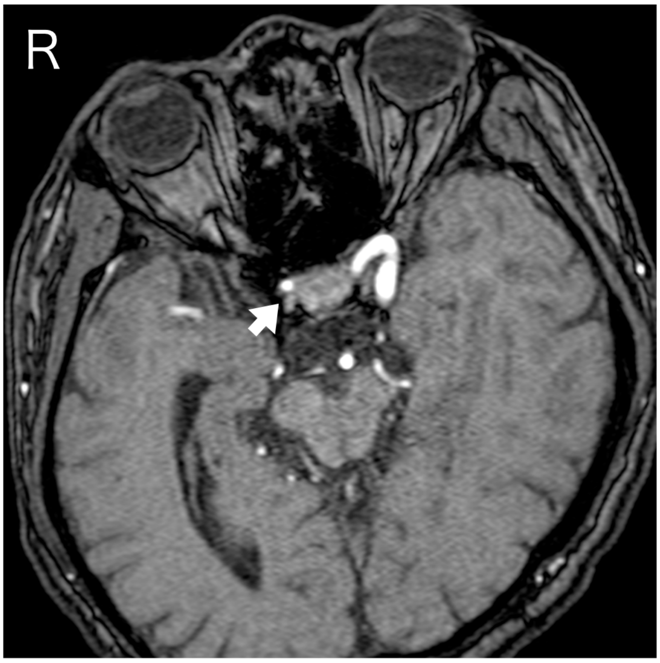

Supplemental Figure 1-1. Time-of-flight magnetic resonance angiography axial source image findings in patient 1 indicating hypoplasia of cavernous portion right internal carotid artery (white arrow).

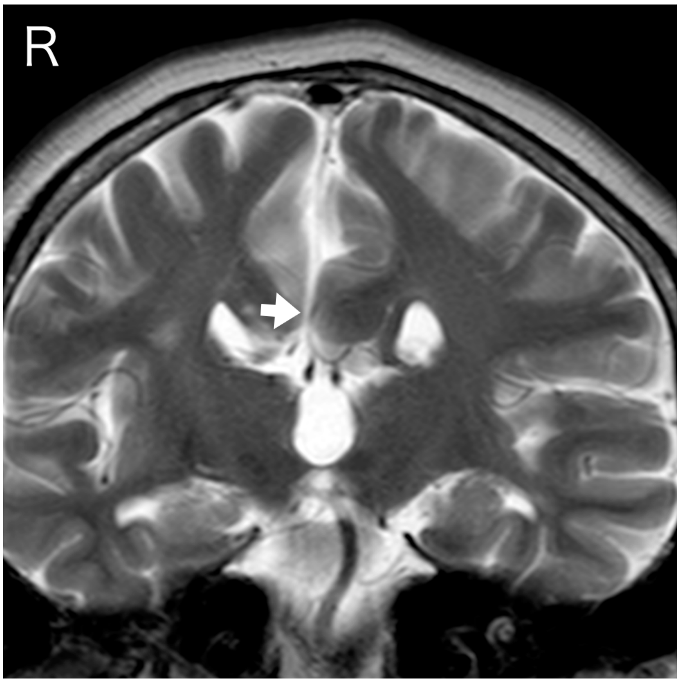

Supplemental Figure 1-2. Coronal image of T2 weighted magnetic resonance image findings in patient 1 indicating hypoplasia of corpus callosal (white arrow).

Case 2 :

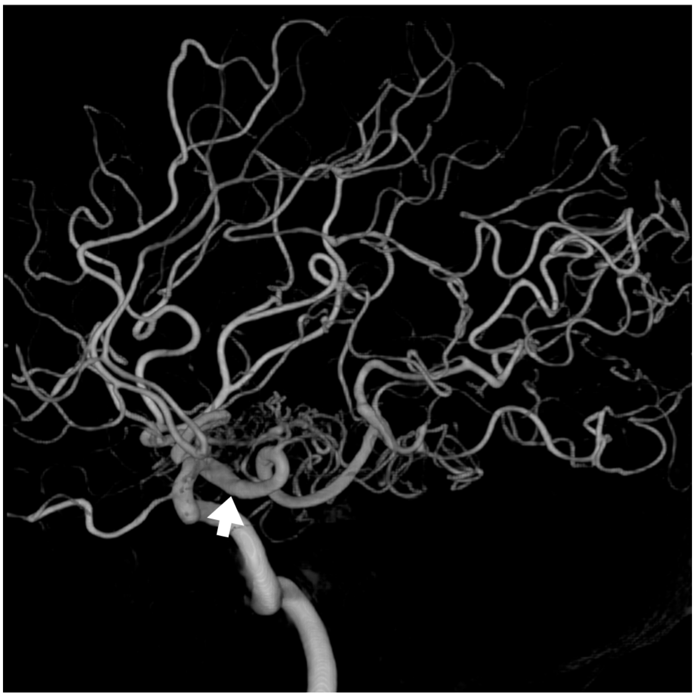

Supplemental Figure 2-1: Lateral view of three-dimensional digital subtraction right internal carotid angiographical findings in patient 2 indicating fatal type posterior communicating artery (white arrow).

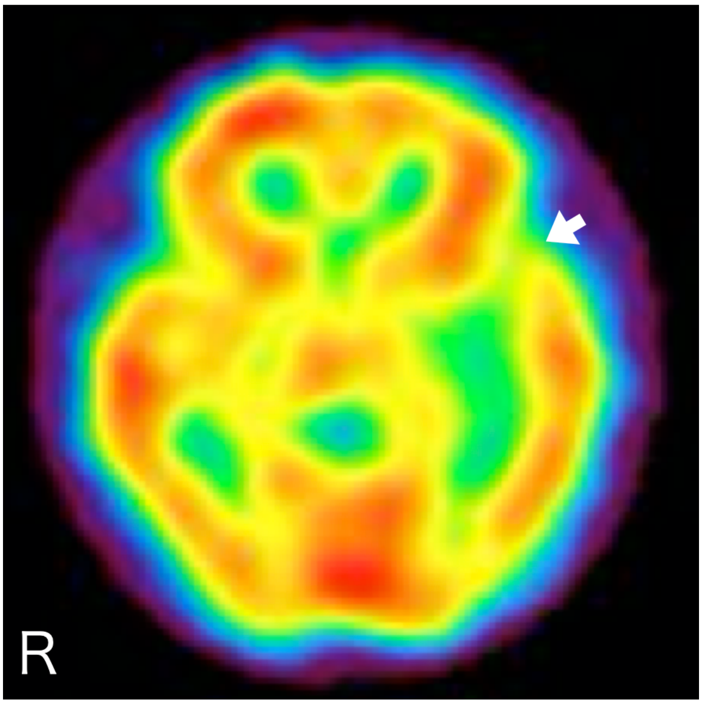

Supplemental Figure 2- 2: IMP single-photon emission computed tomographic findings of patient 2 indicating slight low perfusion area in left middle cerebral artery territory (white arrow).

Case 3:

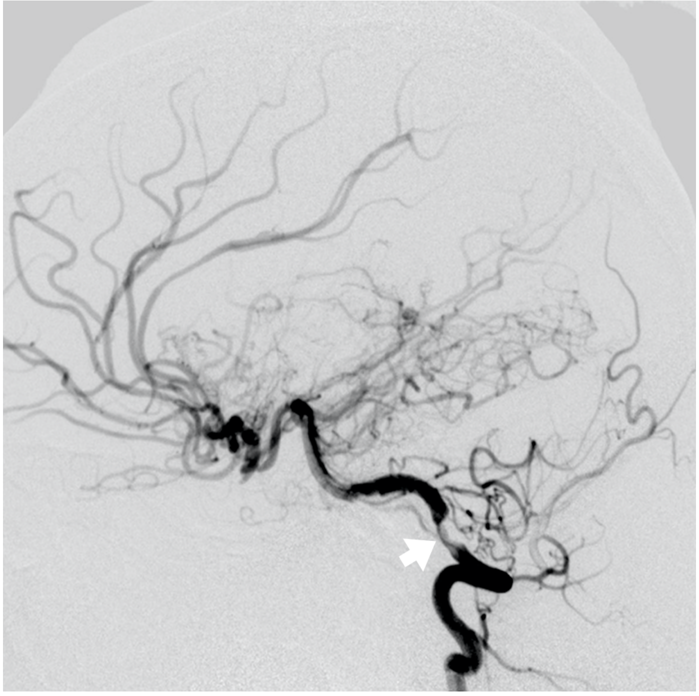

Supplemental Figure 3-1. Lateral view of digital subtraction left vertebral angiographical findings in patient 3 indicating left vertebral artery segmental hypoplasia (white arrow).

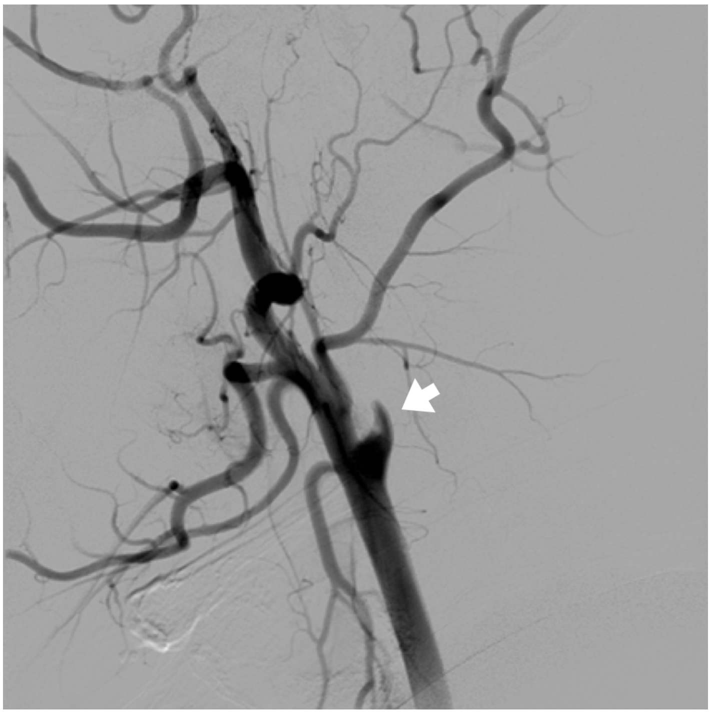

Supplemental Figure 3-2. Lateral view of digital subtraction right common carotid angiographical findings in patient 3 indicating dysplasia (white arrow) of the internal carotid artery.

Case 4:

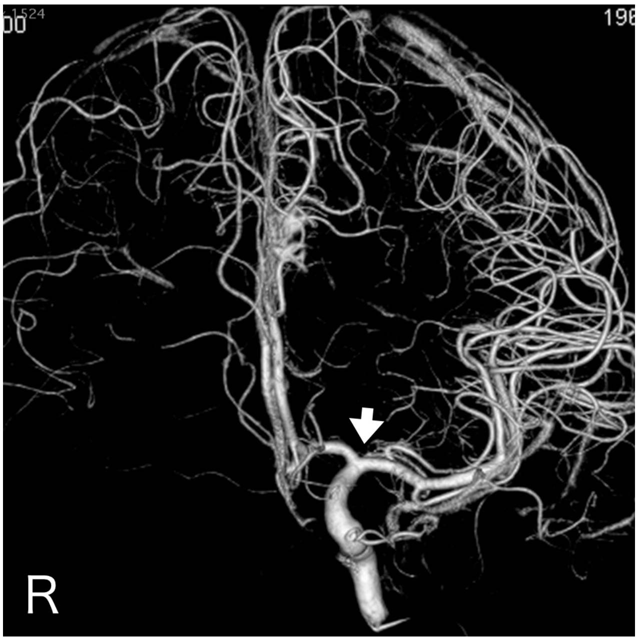

Supplemental Figure 4-1. Anterior-posterior view of three-dimensional digital subtraction left internal carotid angiographical findings in patient 4 indicating no stenotic area and moyamoya vessels in the terminal portion of the internal carotid artery or middle cerebral artery (white arrow). Based on these radiographic findings, this is not moyamoya disease

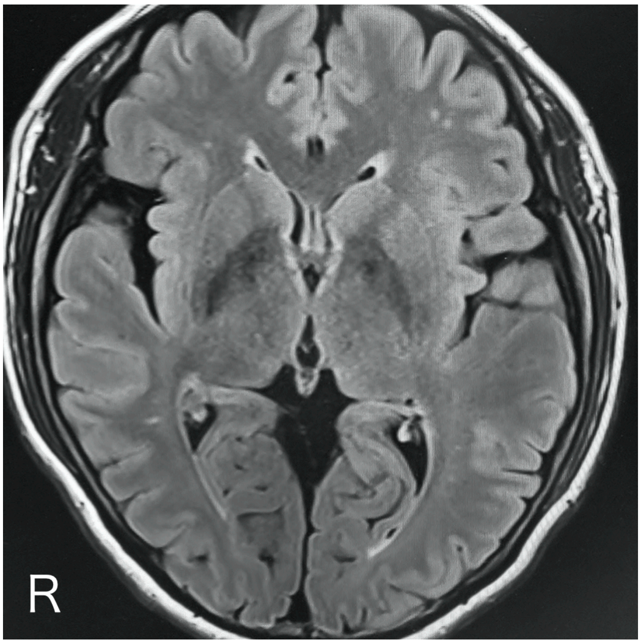

Supplemental Figure 4-2. Fluid-attenuated inversion recovery magnetic resonance images show no abnormal blood vessels in the basal ganglia, ivy sign in the cerebral cortex, or ischemic legion in the right side.

Case 5 :

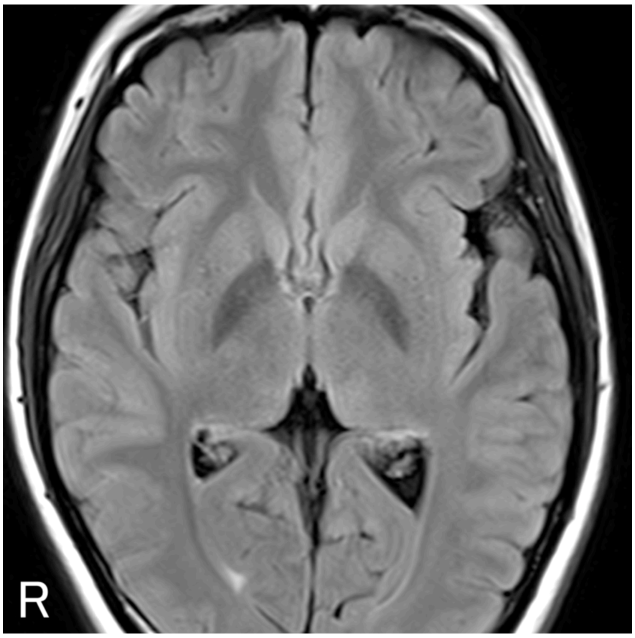

Supplemental Figure 5-1. Fluid-attenuated inversion recovery magnetic resonance images show no abnormal blood vessels in the basal ganglia, ivy sign in the cerebral cortex, or ischemic legion in the right side.

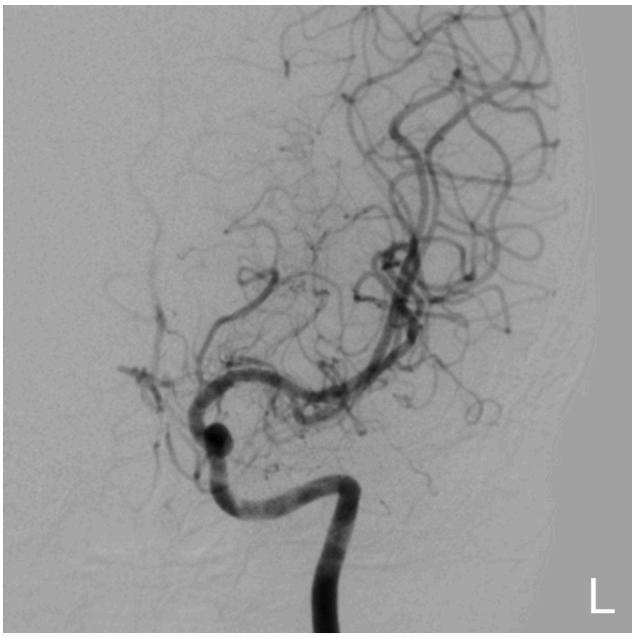

Supplemental Figure 5-2. Anterior-posterior view of left digital subtraction internal carotid angiographical findings in patient 5 indicating no stenotic area and abnormal collateral vessels, which indicate moyamoya disease.

Case 6:

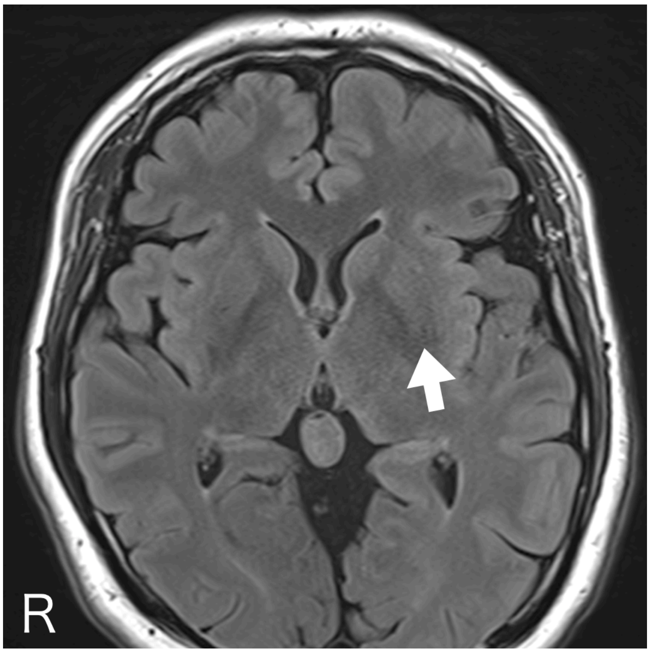

Supplemental Figure 6-1. Fluid-attenuated inversion recovery magnetic resonance images show no abnormal blood vessels in the basal ganglia, ivy sign in the cerebral cortex, or ischemic legion in the left side(white arrow). These radiographic findings do not indicate moyamoya disease.

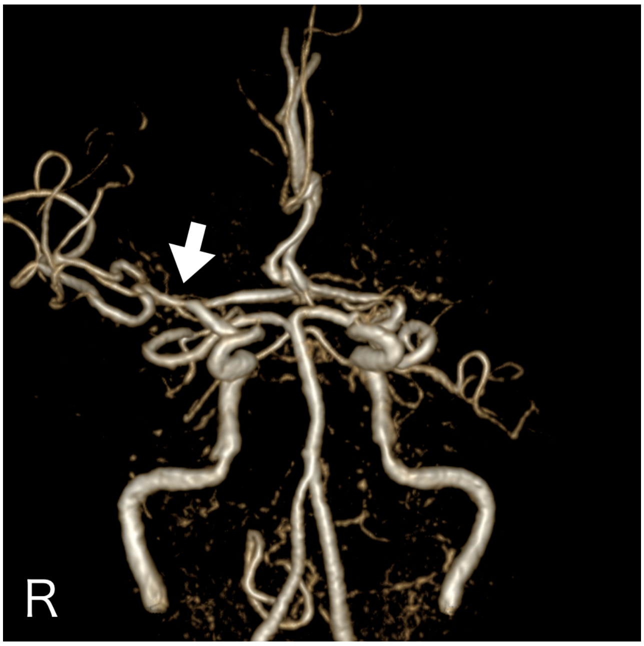

Supplemental Figure 6-2. Anterior-posterior view of magnetic resonance angiography findings in patient 6 indicating dysplasia of left middle cerebral artery and normal right middle cerebral artery(white arrow).

Case 7:

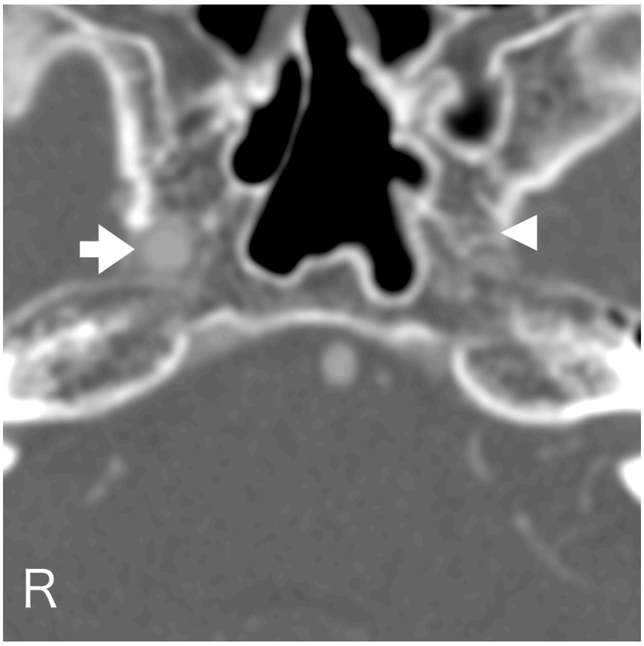

Supplemental Figure 7-1. Post-contrast bone window computed tomographic image findings in patient 7 indicating aplasia of the left carotid canal (white arrowhead) and normal right carotid canal (white arrowhead).

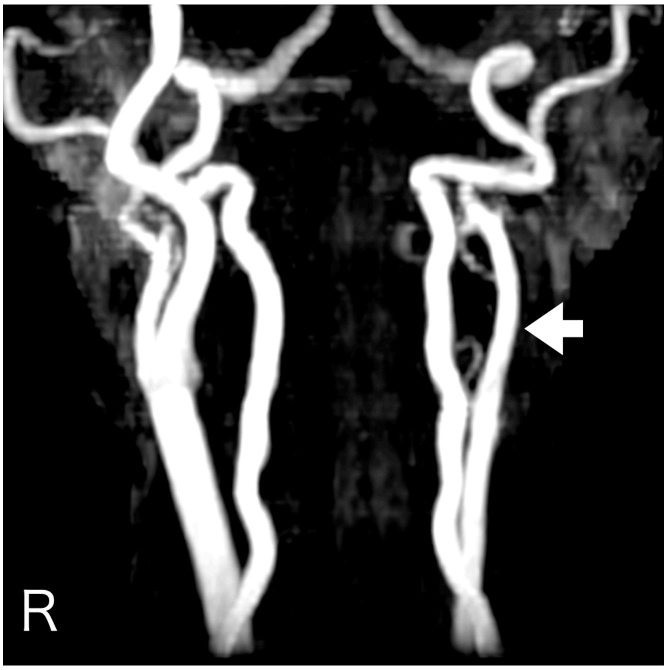

Supplemental Figure 7-2. Cervical anterior-posterior view of magnetic resonance angiography findings in patient 7 indicating aplasia of the left internal carotid artery (white arrow).

Case 8:

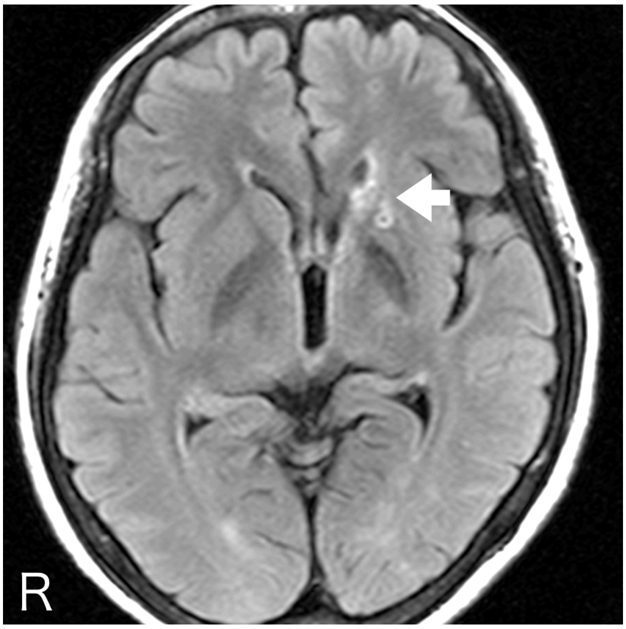

Supplemental Figure 8-1. Fluid-attenuated inversion recovery magnetic resonance images show no abnormal blood vessels in the basal ganglia, ivy sign in cerebral cortex. Ischemic legion in the left caudate head was shown side(white arrow).

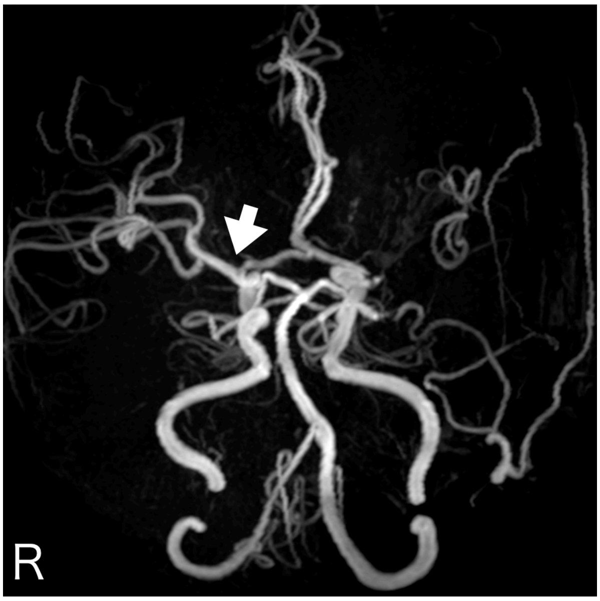

Supplemental Figure 8-2. Anterior-posterior view of magnetic resonance angiography findings in patient 8 indicating dysplasia of left M 1 segment of the middle cerebral artery and normal right middle cerebral artery(white arrow).

Case 9:

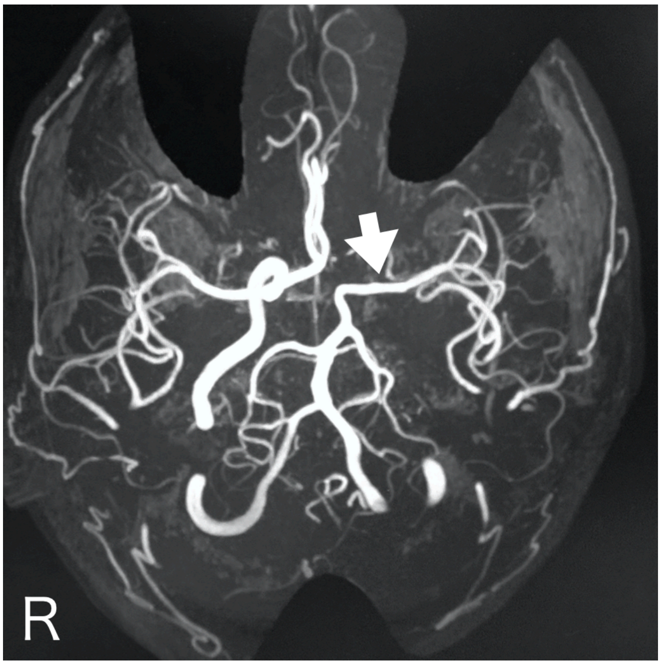

Supplemental Figure 9-1. Anterior-posterior view of magnetic resonance angiography findings in patient 9 indicating hypoplasia of left internal carotid artery and left middle cerebral artery flowed from the vertebral artery (white arrow).

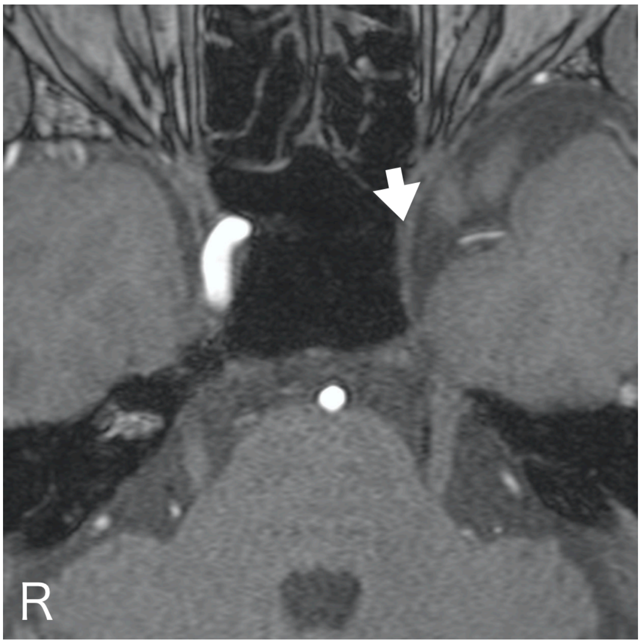

Supplemental Figure 9-2. Time-of-flight magnetic resonance angiography axial source image findings in patient 9 indicating hypoplasia of cavernous portion left internal carotid artery (white arrow).
